# Metabolic profiling of root exudates from two ecotypes of *Sedum alfredii* treated with Pb based on GC-MS

**DOI:** 10.1038/srep39878

**Published:** 2017-01-04

**Authors:** Qing Luo, Shiyu Wang, Li-na Sun, Hui Wang

**Affiliations:** 1 Key Laboratory of Regional Environment and Eco-Remediation of Ministry of Educatione, College of Environment, Shenyang University, Shenyang 110044, China

## Abstract

Phytoremediation is an effective method to remediate Pb-contaminated soils and root exudates play an important role in this process. Based on gas chromatography-mass spectrometry (GC-MS) and metabolomics method, this study focuses on the comparative metabolic profiling analysis of root exudates from the Pb-accumulating and non-accumulating ecotypes of *Sedum alfredii* treated with 0 and 50 μmol/L Pb. The results obtained show that plant type and Pb stress can significantly change the concentrations and species of root exudates, and fifteen compounds were identified and assumed to be potential biomarkers. Leaching experiments showed that l-alanine, l-proline and oxalic acid have a good effect to activate Pb in soil, glyceric acid and 2-hydroxyacetic acid have a general effect to activate Pb in soil. 4-Methylphenol and 2-methoxyphenol might be able to activate Pb in soil, glycerol and diethyleneglycol might be able to stabilize Pb in soil, but these activation effect and stabilization effect were all not obvious.

In response to soil heavy metal pollution, timely treatment and remediation is essential. Phytoremediation, as an emerging green and *in- situ* remediation technology, is widely used to remediate heavy metal contaminated soils. This technology uses plants to remove pollutants from the environment or to render them harmless, including phytoextraction, phytodegradation, rhizofiltration, phytostabilization and phytovolatilization^1^,^2^,^3^. For Pb-contaminated soils, phytoextraction and phytostabilization are the main remediation technologies used. Accumulator plant, species which have the ability to take up heavy metals from the soil to a level higher than the substrate soil, is a key factor for the phytoremediation of heavy metals^4^. Root exudates are important to the mechanisms of how accumulator species absorb, accumulate and tolerate heavy metals.

Root exudates are plant metabolites that are released to the root surface or into the rhizosphere to enhance plant nutrient uptake or cope with environmental- stress, and include low- molecular- weight organic acids, amino acids, sugars, amides, aliphatic acids, aromatic acids, vitamins, peptides, proteins, enzymes, plant hormones, alcohols, ketones, olefins, urea and phytoalexins^5^,^6^,^7^,^8^. The composition and quantity of root exudates vary from plant to plant with two very important factors. One is the plant’s inherent biology, such as plant species and growth cycle; the other is the external environment of plant growth, i.e. soil and its elemental content^9^.

Root exudates can modify the pH and redox potential (Eh) of the rhizosphere; chelate, complex and deposit heavy metals and alter the number and activity of rhizosphere microbes. Through these processes, root exudates can change the chemical speciation of heavy metals and increase or decrease their bio-availability^10^. Low- molecular- weight organic acids (LMWOAs) have a strong ability to bind with heavy metals^11^,^12^,^13^,^14^. For example, citric acid and oxalic acid can enhance the translocation of Cd, Cu and Pb from roots to shoots^15^,^16^. In addition, it has also been shown that dissolved organic matter (DOM), derived from the rhizosphere of *Sedum alfredii*, can significantly reduce Zn and Cd sorption and increase metal mobility through the formation of soluble DOM-metal complexes^17^. However, the variations in root exudates between accumulator and non-accumulator plant species under different stresses which based on an approximate global analysis have rarely been reviewed.

Metabolic profiling is one of the most pragmatic approaches of metabolomics research strategies; it aims at extracting, separating and analyzing a spectrum of metabolites as broad as possible from complex matrices in an effective and reproducible way^18^. It reflects the different metabolic states of the organism and identifies specific biomarkers to describe and distinguish between different states of biological systems^19^. Our group has used metabolic profiling analysis, based on GC-MS technology, to research the variation of root exudates from the Cd hyperaccumulator *S. alfredii* under different Cd exposure concentrations and times, and to identify potential biomarkers^20^,^21^.

Based on GC-MS technology, the present study focused on the metabolic profiling analysis of root exudates from the Pb-accumulating and non-accumulating ecotypes of *Sedum alfredii* treated with 0 and 50 μmol/L Pb to find potential biomarkers associated with accumulation or toleration. And the function of some potential biomarkers was preliminarily discussed by using leaching experiment.

## Results

### Plant growth and Pb accumulation

**Figure 1:**
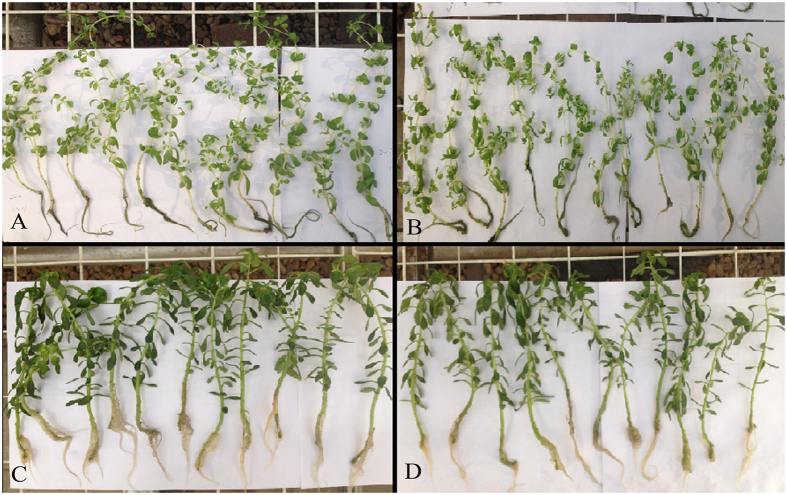
The growth of plant. (**A**) NAE under 0 μmol/L Pb treatment; (**B**) NAE under 50 μmol/L Pb treatment; (**C**) AE under 0 μmol/L Pb treatment; (**D**) AE under 50 μmol/L Pb treatment.

In hydroponic experiments, the accumulating ecotype (AE) showed better growth, an erect stem and thicker dark green leaves, while the non-accumulating ecotype (NAE) showed small, thin, and light green leaves. Lead did not significantly affect the growth and appearance of the shoot of the accumulating ecotype when treated with 50 μmol/L Pb. For the non-accumulating ecotype, Pb significantly inhibited plant growth (dry weight, DW) and the color of leaves changed from light green to dark green as the Pb concentration in the solution increased from 0 to 50 μmol/L (Fig. 1).

The amount of Pb accumulated by most plant species is very limited^22^. The accumulating ecotype of *S. alfredii*, however, was shown to accumulate appreciable quantities of Pb. When treated with 50 μmol/L Pb, the Pb concentrations in the shoots and roots of the accumulating ecotype were, respectively, 136.8 mg/kg (DW) and 893.7 mg/kg (DW). Compared to the accumulating ecotype, the non-accumulating ecotype accumulated less Pb with Pb concentration of 73.9 mg/kg (DW) in the shoots and 2510.9 mg/kg (DW) in the roots (Table 1).

The root- to- shoot transfer of Pb in plants is usually low^22^,^23^. When Pb enters the plant root, it encounters the neutral pH, high phosphate, and high carbonate environment of the intercellular spaces. Under these conditions, Pb precipitates as phosphate or carbonate and does not reach the xylem for translocation^24^,^25^. However, a higher transfer coefficient (shoot/root ratio of heavy metal concentration in plant) is important for the practical phytoremediation of heavy metal-contaminated soils. It has been shown that Pb hyperaccumulators usually have a higher transfer coefficient (0.04–0.10) than non-hyperaccumulators species^26^. In this study, the transfer coefficient of the accumulating ecotype treated with 50 μmol/L Pb was 0.15, while that of the non-accumulating ecotype was 0.03, thus, demonstrating that the accumulating ecotype was more effective in transporting Pb from the roots to the shoots than the non-accumulating species (Table 1).

### Metabolic profiling by GC-MS

**Figure 2:**
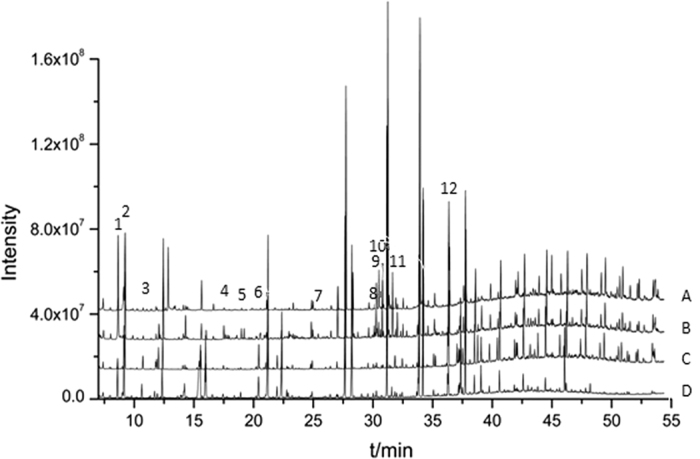
GC-MS total ions chromatogram of root exudates from the non-accumulating and accumulating ecotypes of *S. alfredii*. (**A**) NAE under 0 μmol/L Pb treatment; (**B**) NAE under 50 μmol/L Pb treatment; (**C**) AE under 0 μmol/L Pb treatment; (**D**) AE under 50 μmol/L Pb treatment. Some identified compounds: 1. 2-hydroxyacetic acid-2TMS; 2. lactic acid-2TMS; 3. 3-hydroxypropanoic acid-2TMS; 4. succinic acid-2TMS; 5. nonanoic acid-1TMS; 6. decanoic acid-1TMS; 7. xylose-1MEOX-4TMS; 8. D-pinitol-5TMS; 9. fructose-1MEOX-5TMS; 10. glucose-1MEOX-5TMS; 11. mannitol-6TMS; 12. octadecanol-1TMS.

Fifty-six compounds were detected and identified in metabolic profiling by GC-MS (Fig. 2). The relative contents of these fifty-six compounds were listed in the Supplemental file (Supplemental file 1: Supplemental Table 1).

### Pattern recognition analysis of metabolic profiling

We pursued two multivariate data analysis approaches to differentiate metabolic changes based on the identified root exudates and identify potential biomarkers; an unsupervised method based on principal component analysis (PCA) and a supervised method based on orthogonal partial least-squares discrimination analysis (OPLS-DA). In this work, PCA was firstly performed to classify the metabolic phenotypes.

**Figure 3:**
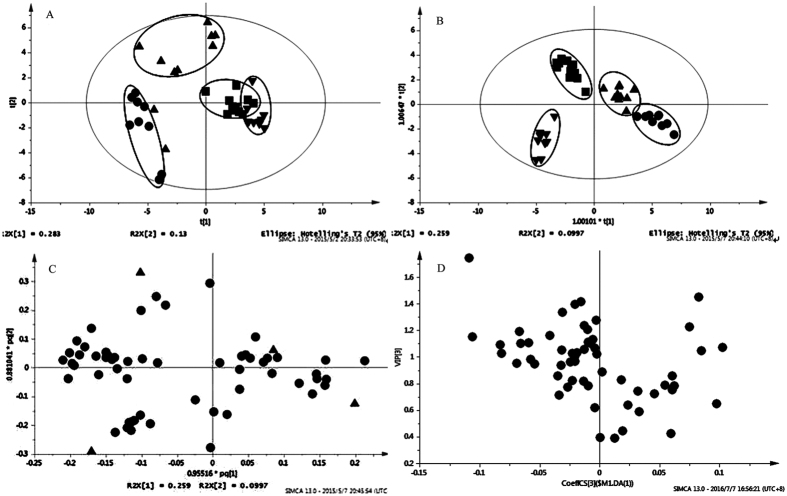
Multivariate analysis of root exudates of the two ecotypes. (**A**) PCA scores plots, (●) NAE under 0 μmol/L Pb treatment, (▲) NAE under 50 μmol/L Pb treatment, (■) AE under 0 μmol/L Pb treatment, (▼) AE under 50 μmol/L Pb treatment; (**B**) OPLS-DA scores plots; (**C**) Loading plot of OPLS-DA; (**D**) VIP score plot from OPLS-DA.

In the PCA score plot (Fig. 3A), a single data point represents a sample, and points clustered together have a more similar biochemical makeup than that of points located apart^27^. The score plot demonstrated a sharp separation amongst the different ecotypes under different Pb stress, suggesting that the plant species and Pb stress can significantly influence the composition and quantity of the root exudates. However, the group of the AE treated with 50 μmol/L Pb was found to be close to the group of the AE treated with 0 μmol/L Pb, which indicates that a low dose of Pb exerted little influence on the AE.

OPLS-DA was then used to maximize the difference between the groups. As showed in Fig. 3B, different groups have a clear separation, indicating that the variation of root exudates caused by plant species and Pb stress.

### Identification of potential biomarkers

**Figure 4:**
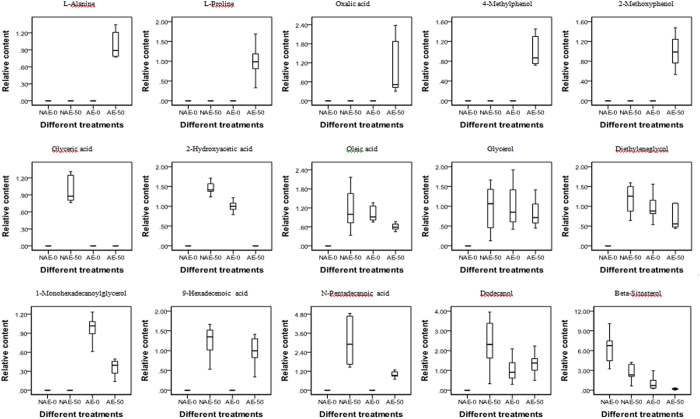
Box and whisker plot. NAE-0, NAE under 0 μmol/L Pb treatment; NAE-50, NAE under 50 μmol/L Pb treatment; AE-0, AE under 0 μmol/L Pb treatment; AE-50, AE under 50 μmol/L Pb treatment.

The use of loading and variable importance in the projection (VIP) score plots in combination can detect differentiating metabolites and potential biomarkers. The loading (Fig. 3C) and VIP plots (Fig. 3D) generated after OPLS-DA processing were applied to interpret the metabolic pattern, and visually show the weight of mass spectral signals attributed to the clustering and discrimination observed in the scores plots. The threshold of VIP values was set to 1.0. An analysis of variance (ANOVA, *p* < *0.05*) was further performed to assess the statistical validity of the multivariate analysis and select potential biomarkers. Finally, fifteen compounds were detected and assumed as potential biomarkers (Fig. 4).

As can be seen from the Fig. 4, l-alanine, l-proline, oxalic acid, 4-methylphenol and 2-methoxyphenol were only secreted when the AE was under Pb stress, but glyceric acid was only secreted when the NAE was under Pb stress. Under Pb treatments, the secretions of 2-hydroxyacetic acid, oleic acid, glycerol, diethyleneglycol, and 1-monohexadecanoylglycerol were increased in NAE, but were reduced from AE. Under Pb treatment, the secretions of 9-hexadecenoic acid, n-pentadecanoic acid and dodecanol increased, while the secretion of beta-sitosterol decreased for both the AE and the NAE.

### Function of potential biomarkers

For further researching the function of potential biomarkers, a series of leaching experiment were performed. Because of oleic acid, 1-monohexadecanoylglycerol, 9-hexadecenoic acid, n-pentadecanoic acid, dodecanol and beta-sitosterol are poorly soluble or insoluble in water, the leaching experiments of these compounds were not conducted. In addition, the concentration of root exudates such as organic acid, amino acid around rhizosphere is low, so a series low concentration of leaching experiment were carried out.

It can be seen from Table 2 that l-alanine and l-proline could activate Pb in soil, and the activation effect increased with the increase of the concentration of l-alanine and l-proline. The highest activation rate was 3.218% and 2.795%, respectively. When the concentration was 2 mmol/L, the extraction of oxalic acid was lower than the control (0 mmol/L). But when the concentration increased to 4 mmol/L, the extraction was obviously higher than the control, and the activation effect increased with the increase of the concentration of oxalic acid. The highest activation rate was 4.618%. 4-Methylphenol and 2-methoxyphenol might be able to activate Pb in soil, but the activation effect was not obvious, the highest activation rate was merely 0.243% and 0.256%, respectively. Glyceric acid and 2-hydroxyacetic acid also could activate Pb in soil, only the activation effect was lower, and obviously lower than the activation effect of oxalic acid, l-alanine and l-proline. The extraction of glycerol and diethyleneglycol were all lower than the control, may be these two compounds could stabilize Pb in soil, but these stabilization effects were not obvious.

## Discussion

The ability of plants to tolerate and accumulate heavy metals is very important for phytoremediation. As an accumulator, it first needs to be resistant to many heavy metals. Secondly, it needs to solubilize heavy metals around the rhizosphere and lastly, the plant must absorb heavy metals in the over-ground part of plants. In these processes, root exudates play an important role^28^,^29^.

Root exudates have many different kinds, can change quickly, and are influenced by many factors, such as soil structure, plant species, environmental- stress, and so on refs 6,30. In this study, fifty-six compounds from the two ecotypes of *S. alfredii* were detected and identified, though there were many chromatographic peaks which were not identified. Based only on these fifty-six compounds, obvious differences in quantity or composition of the root exudates released from the two ecotypes of *S. alfredii* under Pb stress were detected by PCA and OPLS-DA.

In this study, fifteen compounds resulted in an obvious separation between different treatments were identified based on the loadings plot and the VIP values of OPLS-DA and ANOVA. We speculate that these fifteen compounds might play a main role in the process of *S. alfredii* to tolerate or accumulate Pb. This role may be to mobilize or stabilize Pb.

Leaching experiments showed that l-alanine, l-proline and oxalic acid have a good effect to activate Pb in soil. Glyceric acid and 2-hydroxyacetic acid could activate Pb in soil, but the activation effect is poorer. 4-Methylphenol and 2-methoxyphenol might be able to activate Pb in soil, glycerol and diethyleneglycol might be able to stabilize Pb in soil, but these activation effect and stabilization effect were all not obvious.

Previous researches have demonstrated that organic acids and amino acids play an extremely important role in the process of how accumulator species tolerate and accumulate heavy metals^31^,^32^,^33^. Oxalic acid can potentially liberate Pb from pyromorphite in contaminated soils^34^. Debela *et al*. concluded that the abundance of oxalic acid and citric acid in soil environments can be one factor contributing to the poor efficiency of P amendment practices to effectively immobilize Pb and Zn in metal contaminated soils^35^. Cui *et al*. considered that 1.2 mmol/L oxalic acid significantly (P < 0.05) increased Pb uptake when seeds (*Zinnia elegans* Jacq.) were treated with 2.4 mmol/L Pb^36^. In this study, oxalic acid was selected as the potential biomarker and has a good effect to activate Pb in soil. When the pH of the system was high, oxalic acid could form insoluble Pb-oxalate complexes on the soil surface with Pb^37^. So under the low concentration, the extraction of oxalic acid was lower than the control.

Organic acids could reduce the pH around the rhizosphere, and then activated the insoluble minerals in soils, improved the bioavailability of heavy metals^38^,^39^. In this study, glyceric acid and 2-hydroxyacetic acid were selected as the potential biomarker and could activate Pb in soil. Because of glyceric acid and 2-hydroxyacetic acid are weak acid, the activation effect of these two compounds were general.

L-proline and l-histidine may increase Cd accumulation by *Solanum nigrum*^40^. L-cysteine has been shown to form neutral stable complexes with Pb and decrease its sorption on palygorskite and sepiolite; whereas l-histidine formed cationic soluble His–Pb complexes which were slightly adsorbed by the surfaces of sepiolite via electrostatic attraction and reduced the Pb sorption capacity of sepiolite^41^. In this study, l-alanine and l-proline were selected as the potential biomarkers and have a good effect to activate Pb in soil.

The hydroxyl or OH-phenolic groups could complexing react with heavy metal ions in soil, formed a stable complex compound and reduced the activity of heavy metal ions^42^. But Mench *et al*. pointed out that organic acids, amino acids and phenolic compounds could react with heavy metal ions to form soluble compounds, and then improve the bioavailability of heavy metals^43^. Glycerol and diethyleneglycol have hydroxyl group, so these two compounds might be able to stabilize Pb in soil. 4-Methylphenol and 2-methoxyphenol are phenolic compounds which have OH-phenolic group, so the roles of these two compounds are complicated. In this study, glycerol and diethyleneglycol might be able to stabilize Pb in soil, 4-methylphenol and 2-methoxyphenol might be able to activate Pb in soil, but these activation effect and stabilization effect were all not obvious.

The function of other potential biomarkers, such as 9-hexadecenoic acid, n-pentadecanoic acid, and so on, were not discussed in this study, because of these compounds are poorly soluble or insoluble in water. The way which root exudates activate heavy metals in soil have many, such as the indirect activation of microbes^44^, these compounds maybe used this approach. We will research these in the further.

Based on the GC-MS and metabolomics results, this study has focused on the comparative metabolic profiling analysis of root exudates from the Pb-accumulating and non-accumulating ecotypes of *Sedum alfredii* under Pb stress. The results obtained show that plant type and Pb stress can significantly change the levels and species of root exudates, fifteen compounds were identified and assumed as potential biomarkers. Through leaching experiments, the functions of some potential biomarkers were discussed. L-alanine, l-proline and oxalic acid have a good effect to activate Pb in soil, glyceric acid and 2-hydroxyacetic acid have a general effect to activate Pb in soil. 4-Methylphenol and 2-methoxyphenol might be able to activate Pb in soil, glycerol and diethyleneglycol might be able to stabilize Pb in soil, but these activation effect and stabilization effect were all not obvious.

## Methods

### Chemicals and reagents

Methanol (HPLC grade) was bought from Fisher (USA). Methoxamine hydrochloride and N-methyl-N-(trimethylsilyl) trifluoracetamide (MSTFA) were obtained from Sigma (USA). Compositions of the nutrient solution, Pb(NO_3_)_2_, NaOH, HCl, NaNO_3_, l-alanine, l-proline, oxalic acid, glyceric acid, 2-hydroxyacetic acid, glycerol, diethyleneglycol, 4-methylphenol and 2-methoxyphenol (analytic grade) and pyridine (HPLC grade) were purchased from Sinopharm Chemical Reagent Co., Ltd. (ShangHai, China).

### Plant culture

Seedlings of *S. alfredii* were cultivated hydroponically. Accumulating ecotype of *S. alfredii* (AE) was collected from an old Pb/Zn mining area in Quzhou City, Zhejiang Province, China. Non-accumulating ecotype of *S. alfredii (Sedum Sarmentoum Bunge*) (NAE) was obtained from suburb of Shenyang in Liaoning Province of China. After having collected the plants, they were grown in non-contaminated soil in Shenyang University for several generations to minimize the internal metal contents. Uniform and healthy shoots were selected and cultivated in a basal nutrient solution containing 3.0 mmol/L KNO_3_, 0.5 mmol/L NH_4_H_2_PO_4_, 2.0 mmol/L Ca(NO_3_)_2_, 1.0 mmol/L MgSO_4_·7H_2_O, 4.5 μmol/L MnCl_2_·4H_2_O, 23 μmol/L H_3_BO_3_, 0.4 μmol/L ZnSO_4_·7H_2_O, 0.15 μmol/L CuSO_4_·5H_2_O, 0.05 μmol/L H_2_MoO_4_·H_2_O, and 22 μmol/L EDTA-Fe. The nutrient solution pH was adjusted to 6.0 using 0.1 mol/L NaOH or HCl every day. Plants were grown under greenhouse conditions with natural light and the temperature varied from 10 to 20 °C. The nutrient solution was continuously aerated and renewed every 4 days.

### Pb treatment

Intact roots of two week old seedlings of *S. alfredii* were rinsed in deionized water, and then transferred to custom-built hydroponic vessels (one seedling in each 300 mL vessel) containing Pb uptake solution. In addition to the nutrient solution components, the uptake solution contained 0 or 50 μmol/L Pb(NO_3_)_2_. In order to prevent Pb precipitation, the NH_4_H_2_PO_4_ concentration in nutrient solution was adjusted to 5 μmol/L. Each treatment was replicated eleven times. There were 44 pots (1 plant per pot) in total.

### Collection of root exudates and plants

The procedure for the collection of root exudates has been described by Hao *et al*.^45^ After growing for 4 days in the nutrient solution spiked with Pb, the plants were transplanted to sterilized pots with 50 mL deionized water to collect the root exudates for 6 h. After collection, the plant roots in each pot were washed with 100 mL of deionized water and immersed in 20 mmol/L Na_2_-EDTA (disodium ethylenediaminetetraacetate) for 15 min to remove Pb adhering to the root surfaces^46^. Then the roots and shoots were harvested separately.

### Pretreatment of root exudates and plant

The root exudates from each pot were frozen in liquid nitrogen and freeze-dried for 2 days. The dried residue was resuspended in 100 mL of deionized water and freeze-dried again, then redissolved in 10 mL of cold MeOH. The sample was blown to dryness under a gentle nitrogen flow, and reconstituted in 1 mL of *n*-hexane. Then, samples were derivatized by 40 μL methoxyamine hydrochloride (20 mg/mL in pyridine, 2 h, 37 °C) and 70 μL N-methyl-N-(trimethylsilyl) trifluoroacetamide (MSTFA) (30 min, 37 °C), for GC-MS analysis. Refer to some previous studies for more information on this method^47^,^48^.

The fresh shoots and roots were washed with deionized water, air-dried to remove the water adhering to them, dried at 70 °C for 72 h, and then their dry weight was determined. The dried plant samples were powdered and digested using the HNO_3_-HClO_4_ method and the Pb concentration was determined by atomic absorption spectroscopy (AAS).

### GC-MS analysis of root exudates

One microliter of each sample was injected into the GC (Thermo Trace GC Ultra-PolarisQ, USA) in the splitless mode. GC separation was conducted on a capillary column TR-5MS (30 m × 0.25 mm × 0.25 μm, Thermo Fisher, USA). The injector temperature was controlled at 230 °C and the split rate of the injector was 1:50. Helium was used as a carrier gas at a constant flow rate of 1.0 mL/min. The initial column temperature was kept at 70 °C for 1 min, then, the temperature was increased from 70 °C to 76 °C at a rate of 1 °C/min, then increased from 76 °C to 330 °C at a rate of 5 °C/min and held there for 10 min. The transfer line temperature and ion-source temperature were controlled at 250 °C and 210 °C respectively. Ionization was achieved using a 70eV electron beam. Mass spectra were recorded from m/z 50 to 600 at a rate of 2 s in full scan mode, and the solvent delay time was 3 min.

### Data processing and multivariate data analysis

The GC-MS data was processed using the automatic mass spectral deconvolution and identification system (AMDIS, version 2.71) and the metabolomics ion-based data extraction algorithm (MET-IDEA, version 2.08). The AMDIS database was a plant metabolites database containing the Fiehn and Golm Metabolome Database (GMD) and the qualitative standard was similarity greater than 70%. The MET-IDEA input was the output of AMDIS, and the other parameters were: (i) chromatography: GC; average peak width, 0.1; minimum peak width, 0.3; maximum peak width, 6; peak start/stop slope, 1.5; adjusted retention time accuracy, 0.95; peak overload factor, 0.3; (ii) mass spec: trap; mass accuracy, 0.1; mass range, 0.5; (iii) AMDIS: exclude ion list, 73, 147, 281, 341, 415; lower mass limit, 50; ions per component, 1. The peak of 9.30 min detected in all of the samples was used for retention time calibration. Subsequently, each peak area of the identified root exudates was normalized according to each compound based on the first appeared. The normalization method was the peak area values divided by the average of the compound which first appeared.

Multivariate data analysis was achieved on the normalized GC-MS datasets with software package SIMCA-P (version 13.0, Umetrics, Sweden). Principal component analysis (PCA) was carried out on the dataset to generate an overview of the sample distribution and observe possible outliers. The orthogonal partial least-squares discrimination analysis (OPLS-DA) was further performed with the unit-variance scaled GC-MS data as X matrix and class information as Y matrix to identify the metabolites that significantly contribute to intergroup differentiation. The OPLS-DA models were validated using a seven-fold cross validation method and the quality of the model was described by the parameters R^2^X and Q^2^ values. The Variable Importance in the Projection (VIP) value (VIP > 1) was used to evaluate the variable contribution and identify the potential biomarkers. The univariate statistical analysis was performed by SPSS 19.0 for further identification of potential biomarkers, including box figure analysis and analysis of variance (ANOVA), and *p*-value was set as 0.05 for statistical significance.

### Leaching experiment

The soil used in this study was collected from the Shenyang Zhangshi Irrigation Area, located in the western suburbs of Shenyang. Soil sample was air dried and sieved through a 2-mm sieve. The physical-chemical characteristics of the soil sample are presented: organic matter, 2.1%; pH, 6.4; CEC (cation-exchanged capacity), 16.8 cmol/kg; clay, 40.1%, loam, 41.3%, sandy, 18.6%. The content of Pb in the soil is 72.4 mg/kg.

A batch experiment was performed with the potential biomarker as an extractant to test the effects of potential biomarker. Three series of plastic tubes were filled with 20 mL NaNO_3_ solution (0.1 mol/L in deionized water) containing various concentrations of one of the potential biomarkers. The concentration was 0, 2, 4, 8, 16 mmol/L, respectively. Each tube received 2 g of soil and was shaken for 3 h at 20 °C, then centrifuged at 7000 rpm for 20 min. The supernatants were filtered through a Whatman NO. 42 paper, and the filtrates were analyzed for Pb by AAS.

## Additional Information

**How to cite this article**: Luo, Q. *et al*. Metabolic profiling of root exudates from two ecotypes of *Sedum alfredii* treated with Pb based on GC-MS. *Sci. Rep.* **7**, 39878; doi: 10.1038/srep39878 (2017).

**Publisher's note:** Springer Nature remains neutral with regard to jurisdictional claims in published maps and institutional affiliations.

## Supplementary Material

Supplementary Table 1

## Figures and Tables

**Table 1:** The dry weights, Pb concentrations and Pb accumulation in various tissues of *S. alfredii* under Pb stress.

Ecotypes	Pb concentration (μmol/L)	Dry weight (n = 11)		Pb concentration (n = 11)			Pb accumulation (n = 11)		
		Shoot	Root	Shoot	Root	Transfer coefficient	Shoot	Root	Shoot/Root
		mg/plant		mg/kg DW		—	μg/plant		—
NAE	0	93.3 ± 6.1	7.6 ± 0.4	8.6 ± 1.2	44.2 ± 3.6	0.19	0.81 ± 0.03	0.34 ± 0.02	2.38
	50	75.6 ± 4.2	6.8 ± 0.6	73.9 ± 5.4	2510.9 ± 118.6	0.03	5.59 ± 0.52	17.1 ± 2.13	0.33
AE	0	143.4 ± 5.5	12.3 ± 0.6	11.2 ± 1.2	78.3 ± 6.5	0.14	1.61 ± 0.24	0.96 ± 0.12	1.68
	50	145.2 ± 7.8	16.8 ± 1.3	136.8 ± 7.9	893.7 ± 18.2	0.15	19.9 ± 2.43	15.1 ± 1.34	1.32

Values are expressed as mean  ±  standard deviation. Transfer coefficient = Shoot Pb concentration/Root Pb concentration. Pb accumulation = Pb concentration × dry weight.

**Table 2:** The effect of potential biomarkers activated Pb in soil (mg/kg).

Potential biomarkers	0 mmol/L	2 mmol/L	4 mmol/L	8 mmol/L	16 mmol/L
l-alanine	0.132 ± 0.003 (0.182)	0.253 ± 0.007 (0.350)	0.654 ± 0.029 (0.904)	1.018 ± 0.034 (1.406)	2.330 ± 0.030 (3.218)
l-proline	0.132 ± 0.003 (0.182)	0.326 ± 0.010 (0.450)	0.548 ± 0.019 (0.756)	1.089 ± 0.021 (1.505)	2.024 ± 0.026 (2.795)
oxalic acid	0.132 ± 0.003 (0.182)	0.063 ± 0.002 (0.087)	0.704 ± 0.006 (0.973)	2.082 ± 0.043 (2.875)	3.343 ± 0.029 (4.618)
4-methylphenol	0.132 ± 0.003 (0.182)	0.113 ± 0.005 (0.156)	0.145 ± 0.005 (0.200)	0.176 ± 0.006 (0.243)	0.157 ± 0.006 (0.216)
2-methoxyphenol	0.132 ± 0.003 (0.182)	0.097 ± 0.005 (0.133)	0.126 ± 0.005 (0.174)	0.146 ± 0.006 (0.202)	0.185 ± 0.004 (0.256)
glyceric acid	0.132 ± 0.003 (0.182)	0.213 ± 0.007 (0.295)	0.312 ± 0.003 (0.431)	0.454 ± 0.006 (0.627)	0.523 ± 0.011 (0.722)
2-hydroxyacetic acid	0.132 ± 0.003 (0.182)	0.191 ± 0.004 (0.264)	0.285 ± 0.005 (0.394)	0.514 ± 0.007 (0.710)	0.788 ± 0.005 (1.089)
glycerol	0.132 ± 0.003 (0.182)	0.113 ± 0.004 (0.156)	0.085 ± 0.003 (0.118)	0.103 ± 0.003 (0.142)	0.126 ± 0.004 (0.174)
diethyleneglycol	0.132 ± 0.003 (0.182)	0.091 ± 0.004 (0.126)	0.083 ± 0.003 (0.114)	0.113 ± 0.003 (0.155)	0.145 ± 0.006 (0.201)

Values in brackets are the percentage of the extraction of potential biomarkers to the total content of Pb in soil.
