# Supplementary material for: Metabolic profiling of root exudates from two ecotypes of Sedum alfredii treated with Pb based on GC-MS
Source: Sci Rep. 2017 Jan 4;7:39878. doi: 10.1038/srep39878 (PMC5209681; doi:10.1038/srep39878)
Supplement: Supplementary Table 1 [file srep39878-s1.pdf]

Title: Metabolic profiling of root exudates from two ecotypes of *Sedum alfredii* treated with Pb based on GC-MS

Qing Luo, Shiyu Wang, Li-na Sun, Hui Wang

Supplemental file 1 Supplemental Table

Supplemental Table legend

Supplemental Table 1 The retention time, name and relative content of the identified compounds of root exudates from *S. alfredii*.

Values are expressed as mean ± standard deviation. MEOX, methoxylation; TMS, trimethylsilylation.

Supplemental Table 1 The retention time, name and relative content of the identified compounds of root exudates from *S. alfredii*

ID	Retention time	Identified compounds	Pb exposed concentration (μmol/L)			
			AE		NAE	
			0 (n=11)	50 (n=11)	0 (n=11)	50 (n=11)
1	6.70	Oxalic acid (2TMS)	1.00±0.06	0.91±0.02	0.86±0.09	1.49±0.05
2	9.07	2-Hydroxyacetic acid (2TMS)	1.00±0.04	0.00±0.00	0.00±0.00	1.38±0.09
3	9.30	Lactic acid (2TMS)	1.00±0.15	2.22±0.33	2.79±1.24	1.44±0.29
4	9.51	Oxalic acid (2TMS)	0.00±0.00	1.00±0.36	0.00±0.00	0.00±0.00
5	9.94	2-Hydroxyacetic acid (2TMS)	1.00±0.18	0.86±0.14	1.64±0.32	1.21±0.23
6	10.73	L-Alanine (2TMS)	0.00±0.00	1.00±0.11	0.00±0.00	0.00±0.00
7	12.21	3-Hydroxypropanoic acid (2TMS)	1.00±0.11	1.32±0.35	1.57±0.16	3.86±2.47
8	12.24	3-Pyridinol (1TMS)	1.00±0.05	4.42±2.82	1.35±0.27	3.84±2.47
9	12.58	4-Methylphenol (1TMS)	0.00±0.00	1.00±0.24	0.00±0.00	0.00±0.00
10	12.64	3-Hydroxybutanoic acid (2TMS)	1.00±0.34	2.09±1.63	1.21±0.35	1.64±0.57
11	14.19	Naphthalene	1.00±0.31	1.28±0.24	2.32±0.66	2.48±0.65
12	14.94	2-Methoxyphenol (1TMS)	0.00±0.00	1.00±0.17	0.00±0.00	0.00±0.00
13	15.32	Diethyleneglycol (2TMS)	1.00±0.14	0.73±0.15	0.00±0.00	1.77±0.61
14	15.75	Urea (2TMS)	1.00±0.39	0.86±0.42	1.21±0.37	0.94±0.21
15	16.02	Phosphoric acid (3TMS)	1.00±0.22	1.09±0.33	0.86±0.15	0.74±0.17

16	16.04	Glycerol (3TMS)	1.00±0.14	0.80±0.10	0.00±0.00	0.95±0.23
17	16.09	Phosphoric acid (3TMS)	1.00±0.42	1.38±0.15	0.95±0.21	3.12±1.01
18	16.18	Phosphoric acid (3TMS)	1.00±0.07	0.85±0.21	1.32±0.35	0.96±0.22
19	17.00	Glycine (3TMS)	1.00±0.69	0.45±0.07	0.65±0.12	0.78±0.19
20	17.47	Succinic acid (2TMS)	1.00±0.11	1.86±0.82	0.79±0.21	1.21±0.17
21	17.70	Glyceric acid (3TMS)	0.00±0.00	0.00±0.00	0.00±0.00	1.00±0.12
22	18.46	L-Alanine (3TMS)	1.00±.62	1.01±0.16	0.85±0.21	0.98±0.25
23	18.80	Nonanoic acid (1TMS)	1.00±0.16	1.47±0.21	0.77±0.15	0.60±0.23
24	21.26	Putrescine (4TMS)	1.00±0.09	1.02±0.05	0.85±0.12	1.23±0.33
25	21.38	Decanoic acid (1TMS)	1.00±0.13	1.31±0.24	0.74±0.11	0.95±0.15
26	22.24	Erythritol (4TMS)	1.00±0.21	0.79±0.15	1.09±0.17	0.86±0.13
27	22.98	L-Proline (2TMS)	0.00±0.00	1.00±0.17	0.00±0.00	0.00±0.00
28	23.32	2,4,6-Tri-tert.-butylbenzenethiol	1.00±0.07	0.82±0.05	1.52±0.13	1.22±0.12
29	24.08	Dodecanol(1TMS)	1.00±0.17	1.34±0.17	0.00±0.00	2.36±0.42
30	25.90	Xylose (4TMS 1MEOX)	1.00±0.24	0.79±0.13	0.94±0.21	1.14±0.19
31	26.11	Dodecanoic acid (1TMS)	1.00±0.27	2.88±1.95	0.00±0.00	1.80±0.68
32	27.63	Putrescine (4TMS)	1.00±0.14	1.21±0.54	0.78±0.21	0.99±0.17
33	28.56	4-Hydroxybenzoic acid (2TMS 1MEOX)	1.00±0.10	0.76±0.21	0.88±0.24	1.13±0.35
34	29.11	Terephthalic acid (2TMS)	1.00±0.48	0.64±0.06	0.00±0.00	0.58±0.21
35	29.73	Phenanthrene	1.00±0.27	0.79±0.15	1.14±0.16	0.67±0.21
36	30.12	D-Pinitol (5TMS)	1.00±0.14	1.56±0.73	3.32±0.85	1.93±0.25

37	30.29	Fructose (5TMS 1MEOX)	1.00±0.72	0.42±0.19	2.99±1.03	4.45±1.78
38	30.36	Tetradecanoic acid (1TMS)	1.00±0.22	0.86±0.08	0.00±0.00	2.04±0.61
39	30.50	Fructose {BP} (5TMS 1MEOX)	1.00±0.85	0.45±0.35	1.96±0.61	2.74±1.26
40	30.80	Glucose (5TMS 1MEOX)	1.00±0.45	0.41±0.14	3.99±1.62	4.13±1.71
41	31.01	1-Methyl-alpha-D-glucopyranoside (4TMS)	1.00±0.34	0.62±0.17	0.85±0.24	1.15±0.89
42	31.18	Glucose (5TMS 1MEOX)	1.00±0.21	0.87±0.23	1.05±0.25	1.17±0.58
43	31.35	Mannitol (6TMS)	1.00±0.17	0.64±0.18	1.00±0.17	0.43±0.06
44	31.66	9-Hexadecenoic acid, methyl ester, (Z)-	1.00±0.22	1.15±0.31	2.06±0.87	0.97±0.14
45	32.05	Hexadecanoic acid, methyl ester	1.00±0.34	2.05±0.51	8.43±3.74	5.61±3.00
46	32.34	N-Pentadecanoic acid (1TMS)	0.00±0.00	1.00±0.08	0.00±0.00	3.07±0.64
47	33.76	9-Hexadecenoic acid(1TMS)	0.00±0.00	1.00±0.20	0.00±0.00	1.29±0.36
48	34.26	Hexadecanoic acid (1TMS)	1.00±0.08	0.82±0.06	0.77±0.19	0.67±0.16
49	36.23	Octadecanol (1TMS)	1.00±0.13	0.90±0.09	5.24±2.35	4.33±1.18
50	37.26	9,12-(Z,Z)-Octadecadienoic acid (1TMS)	1.00±0.16	0.79±0.09	0.00±0.00	1.40±0.36
51	37.36	Oleic acid (1TMS)	1.00±0.29	0.65±0.06	0.00±0.00	1.16±0.23
52	37.82	Octadecanoic acid (1TMS)	1.00±0.08	0.89±0.06	0.71±0.17	0.67±0.12
53	43.23	1-Monohexadecanoylglycerol (2TMS)	1.00±0.10	0.38±0.05	0.00±0.00	0.25±0.05
54	43.42	Sucrose (8TMS)	1.00±0.59	0.48±0.05	1.29±0.26	2.70±1.56
55	44.98	Trehalose (8TMS)	1.00±0.21	0.54±0.20	6.15±2.89	1.61±0.58
56	53.48	beta-Sitosterol (1TMS)	1.00±0.25	0.31±0.08	7.96±1.76	3.23±0.59

Values are expressed as mean ± standard deviation. MEOX, methoxylation; TMS, trimethylsilylation.
